# *De novo* whole-genome assembly of *Chrysanthemum makinoi*, a key wild chrysanthemum

**DOI:** 10.1093/g3journal/jkab358

**Published:** 2021-10-13

**Authors:** Natascha van Lieshout, Martijn van Kaauwen, Linda Kodde, Paul Arens, Marinus J M Smulders, Richard G F Visser, Richard Finkers

**Affiliations:** Plant Breeding, Wageningen University and Research, Wageningen 6708 PB, The Netherlands

**Keywords:** *Chrysanthemum makinoi*, chrysanthemum, genome assembly, annotation

## Abstract

Chrysanthemum is among the top 10 cut, potted, and perennial garden flowers in the world. Despite this, to date, only the genomes of two wild diploid chrysanthemums have been sequenced and assembled. Here, we present the most complete and contiguous chrysanthemum *de novo* assembly published so far, as well as a corresponding *ab initio* annotation. The cultivated hexaploid varieties are thought to originate from a hybrid of wild chrysanthemums, among which the diploid *Chrysanthemum makinoi* has been mentioned. Using a combination of Oxford Nanopore long reads, Pacific Biosciences long reads, Illumina short reads, Dovetail sequences, and a genetic map, we assembled 3.1 Gb of its sequence into nine pseudochromosomes, with an N50 of 330 Mb and a BUSCO complete score of 92.1%. Our *ab initio* annotation pipeline predicted 95,074 genes and marked 80.0% of the genome as repetitive. This genome assembly of *C. makinoi* provides an important step forward in understanding the chrysanthemum genome, evolution, and history.

## Introduction

As one of the most economically important ornamental crops (Anderson 2007), much time has been invested into understanding *Chrysanthemum morifolium* Ramat. and its related varieties and species. One of the key factors of its success as an ornamental crop is the diversity available in petal colors and flower shapes (Song *et al.* 2018), even though the underlying genomic and molecular basis of the shape traits is still poorly understood. This is partly due to the fact that it is a hexaploid with polysomic inheritance (van Geest *et al.* 2017b).

To begin to understand a hexaploid such as *C. morifolium* Ramat. and its traits, we must first look at the whole genus and research the plant’s origins. The *Chrysanthemum* genus consists of species with a basic number of nine chromosomes but with variable ploidy level, from diploid to decaploid (Wang *et al.* 2014). Native across Eurasia and the northern parts of North America, the genus consists of 40 different species (Liu *et al.* 2012; Liu 2020). More than 10 were originally identified as a potential source material for the domesticated *C. morifolium* Ramat. (Hemsley 1889; Stapf 1933; Dowrick 1952; Ackerson 1967), including *Chrysanthemum* *makinoi* (syn. *D. makinoi*), *Chrysanthemum* *indicum* (syn. *D. indicum*), *Chrysanthemum* *lavandulifolium* (syn. *D. lavandulifolium*), and *Chrysanthemum* *zawadskii* (syn. *D. zawadskii*), predominantly in their hexaploid form. The hexaploid *Chrysanthemum* *vestitum* and tetraploid *C. indicum* were later again suggested as major donors based on comparative morphology, cytology, interspecific hybridization, and molecular systematics (Ma *et al.* 2016). Diploids such as *Chrysanthemum* *nankingense*, *C. lavandulifoium*, and *C. zawadskii* have also repeatedly been identified as possible contributors (Dai *et al.* 2005; Liu *et al.* 2012; Ma *et al.* 2016). To date, no one has come up with a conclusive model for *C. morifolium* Ramat.

*Chrysanthemum* *makinoi* is a wild diploid endemic to Japan. While research has been performed in the past with this diploid species (Tanaka 1960; Tanaka and Shimotomai 1968), no one has attempted to assemble its genome. In fact, to date, of the 40 chrysanthemum species only *C*hrysanthemum *seticuspe* (Hirakawa *et al.* 2019) and *C. nankingense* (Song *et al.* 2018) have whole-genome assemblies. The *C. seticuspe* assembly was made using only short-read sequencing and had a total length of 2.722 Gb, with 354,212 contigs, an N50 of 44,741 bp, and a BUSCO score of 88.8% (Hirakawa *et al.* 2019), while *C. nankingense* was assembled using both long and short reads for a total length of 2.527 Gb, with 24,051 contigs, an N50 of 130,678 bp, and a BUSCO score of 92.7% (Song *et al.* 2018). Generating a more contiguous assembly of these diploids has been difficult as chrysanthemum genomes are very repetitive and heterozygous (Won *et al.* 2018a; Nguyen *et al.* 2020).

Long-read data help resolve the repetitive sequences and allows for more contiguous contigs to be assembled (van Dijk *et al.* 2018), so we proceeded with an approach that combined both long read, short read, and proximity ligation methods to build a truly robust assembly. This assembly, along with its corresponding organelle assemblies and transcriptome, will not only expand our understanding of the diploid *C. makinoi* but also help illuminate the complicated polyploidization story that led to *C. morifolium* Ramat. by providing a robust genomic foundation from which to expand.

## Materials and methods

### Plant material

The *C. makinoi* Matsum. et Nakai or No. JP131333 Ryuunougiku plant, or *C. makinoi* for short, was obtained from the NARO (Tsukuba, Japan) genebank. Cuttings were grown in greenhouses at Wageningen University and Research (WUR-Unifarm) according to standard procedures.

### DNA extraction, library preparation, and sequencing

High molecular weight DNA for long-read sequencing was isolated from fresh young *C. makinoi* leaves using a modified (Bernatzky and Tanksley 1986) protocol. Libraries were prepped using the 1D ligation sequencing kits SQK-LSK108 and SQK-LSK109 (Oxford Nanopore Technologies, Oxford, UK) according to the instructions. The samples were sequenced on an Oxford Nanopore GridION using 40 flow cells and the standard protocol. Adaptors were removed using Porechop (Wick 2018) and reads were filtered using Filtlong (Wick 2019), which removed the worst 10% of reads from the shorter reads.

One sample was also sequenced using four differently sized insert libraries (270, 350, 400, and 500 bp) and 150-bp paired-end reads on an Illumina HiSeq 2500 (GenomeScan, Leiden, The Netherlands). Samples were processed using the NEBNext^®^ Ultra DNA library Prep Kit from Illumina. Genome characteristics were estimated using Jellyfish v2.2.10 (Marçais and Kingsford 2011) k-mer counts and GenomeScope (Vurture *et al.* 2017).

High molecular weight DNA of *C. makinoi* was also sequenced by GenomeScan across eight SMRTcells using a PacBio “Sequel SMRT Cell 1M v2” sequencer. Sample preparation was done based on the “PacBio SMRTbell Express Kit v1” protocol. The final library was selected using the Blue Pippin protocol for fragments larger than 15 kb. Primer and polymerase were attached using the “Sequel Binding and Internal Ctrl Kit2.1” kit and purification was done using the PacBio “Procedure & Checklist—AMPure^®^ PB Bead Purication of Polymerase Bound SMRTbell^®^ Complexes” protocol. Sequencing was performed for 10 h on seven of the cells and 20 h for the remaining cell with the recommended amount of “DNA Internal Control Complex 2.1”. The raw data were assessed with the SMRT Link Analysis server v5.1.0.26367 by GenomeScan.

Four tissues (leaves, stems, floral buds, and flowers) used in the study were obtained from a *C. makinoi* cultivated in a greenhouse under long-day conditions, 20-h light/4-h dark cycle, or under short-day conditions, 11-h light/13-h dark cycle, at Dekker Chrysanten (Hensbroek, The Netherlands). All collected plant tissues were frozen immediately in liquid N_2_ and stored at −70°C until the RNA was extracted and isolated using the RNeasy mini kit (Qiagen, Hilden, Germany) and library prepped using the PCR-cDNA sequencing kit (SQK-PCS109; Oxford Nanopore Technologies) according to the manufacturer’s instructions. The samples were sequenced separately on an Oxford Nanopore GridION using nine flow cells in total, according to the standard protocol. Quality control was done using NanoComp v1.9.2 (De Coster *et al.* 2018) and fastq validator from fastq_utils v0.21.0 (Fonseca and Manning) with duplicate read IDs removed.

### Genome assembly and scaffolding

Nanopore reads were base-called with Guppy v3.2 (Oxford Nanopore Technologies) and filtered to keep only the reads from the “pass” folder (*Q* ≥ 7) that had a length above 20 kb and the “fail” folder (*Q* < 7) with a length over 50 kb. PacBio reads over 30- kb long were also added into this dataset. This combination of long reads was assembled using SMARTdenovo v1.0.0 (Liu *et al.* 2021) with “generate consensus” set to 1. Purge Haplotigs (Roach *et al.* 2018) was then used to flatten regions of heterozygosity into a single consensus sequence. Illumina data were subsequently used in conjunction with ntEdit v0.9 (Warren *et al.* 2019) in mode 2 and with a *K* = 50 for two iterations to polish the contigs. Contiguity was further improved with the use of Hi-C and Chicago proximity ligation methods (Dovetail Genomics, Scotts Valley, USA). Final pseudo-molecule level scaffolding was performed using ALLMAPS v0.9.14 (Tang *et al.* 2015) and an integrated genetic map of hexaploid chrysanthemum (van Geest *et al.* 2017a; see Supplementary Table S1 and Supplementary Figure S1). Some by-hand misassembly corrections, verified with the raw long-read data, were also completed (see Supplementary Figure S2). Contigs that remained unplaced among the nine chromosomes in the final assembly were filtered to remove contaminants and unusually high coverage reads. The final chromosomes were named and numbered following the linkage group assignments in a *C. morifolium* Ramat. cross found in van Geest *et al.* (2017a). Read coverage was assessed using Qualimap bamqc v2.2.1 (Okonechnikov *et al.* 2016) and contigs with no or high coverage (>4x the mean coverage) were removed. Subsequently, contaminantsequences were identified using Centrifuge v1.0.4 (Kim *et al.* 2016) using the NCBI’s viral and bacterial libraries (accessed in November 2019) and removed. The remaining reads were placed into a chromosome zero with N-gaps of 200 bp in between each contig.

Organelles were assembled by extracting Nanopore and Illumina reads that aligned to the available *C. seticuspe* (syn. *C. boreale*) chloroplast (Won *et al.* 2018b) and mitochondria (Won *et al.* 2018c) references using Minimap2 v2.17 (Li 2018) and BWA-MEM v0.7.17-r1198-dirty (Li 2013), respectively. A hybrid assembly was then performed for each organelle using Unicycler v0.4.8 (Wick *et al.* 2017). This resulted in a single, circular scaffold assembly for the chloroplast and multiple circular scaffolds for the mitochondria. Based on a visual inspection of each of the mitochondria scaffolds against known chrysanthemum mitochondria assemblies, scaffold 1 was found to represent the entire sequence and was selected as the full circular assembly of the mitochondria genome.

### Genome analysis and quality assessments

QUAST v5.0.2 (Gurevich *et al.* 2013) was used to determine the basic statistics of the final genome assembly such as total length, N50 and the number of contigs/scaffolds. BUSCO v4.0.5 (Simão *et al.* 2015) and the corresponding set of Embryophyta odb10 universal single-copy orthologs was also used to assess the completeness of the genome.

### Repeat and transcript annotation

Before annotating the assembly, we soft-masked the repetitive sequences using RepeatModeler v2.0.1 (Flynn *et al.* 2020).

Gene prediction was done with the Funannotate v1.7.4 (Palmer 2017) pipeline. First, the Funannotate pipeline was trained using the cDNA long reads, UniProtKB v2020_04 database (Bateman 2019), and the BUSCO eukaryote odb9 protein database (Simão *et al.* 2015), to create the input dataset for the Funannotate predict pipeline. The predict pipeline was then run with standard settings and the GeneMark-ET, Augustus, GlimmerHMM, and Snap algorithms. Afterward, filtering of the *ab initio* gene predictions was done using EVidenceModeler (EVM; Haas *et al.* 2008).

To functionally annotate the predicted models, an initial comparison was done using blastp v2.6.0 (Camacho *et al.* 2009) against the SWISS-PROT v4 database (Bairoch and Apweiler 2000) with a cut-off *e*-value of 1.0E−3, a word size of 6, a maximum number of hits set to 20, and the low complexity filter turned on. To identify the domains within the predicted model sets, InterProScan v5.26 (Jones *et al.* 2014) was used along with the panther v12.0 libraries. Finally, the results were processed by a stand-alone version of Blast2Go (Götz *et al.* 2008) using default settings.

## Results and discussion

### Raw sequence quality

Nanopore sequencing resulted in 443.25 Gb of data with a read N50 of 22.6 kb. After base calling, removing adaptors and filtering for reads over 20 kb in length from the “pass” folder, which had a *Q* score of >7, and for reads over 50 kb in length from the “fail” folder, the dataset had a coverage of approximately 53× (assuming a haploid genome size of 3.1 GB) and consisted of 3,924,770 reads. Illumina HiSeq yielded 113.2, 142.0, 133.7, and 120.0 Gb of raw data for the 270, 350, 400, and 500 bp insert size libraries, respectively. Between 90.5% and 94.6% of reads in each insert size had a quality “*q*” score of greater than or equal to 30. PacBio sequencing resulted in 70 Gb of data with an average subread length of 15.5 kb and an N50 of 24.1 kb. This meant a coverage of approximately 30.6× (assuming a haploid genome size of 3.1 GB).

The nanopore cDNA sequencing resulted in datasets with 4.8–7.9 million reads, an average N50 of 1.2–1.4 kb and between 5.0 and 7.9 Gb total (Table  1).

**Table 1:** Sequencing details of cDNA from different plant organs in *C. makinoi*

Source	Mean read length (b)	Mean read quality	Number of reads	Read length N50 (b)	Total bases
Leaf (short d)	977.0	9.0	7,587,930	1,189	7,413,732,394
Leaf (long d)	973.0	9.4	6,780,899	1,209	6,597,664,366
Calyx	1,040.2	10.0	4,833,397	1,247	5,027,548,849
Flower disk florets	1,012.1	9.5	7,072,131	1,331	7,157,901,095
Flower buds	993.6	9.1	7,917,800	1,256	7,867,489,497
Flower ray florets	1,002.8	9.6	7,000,372	1,250	7,020,311,566
Meristem	1,048.0	10.2	5,075,164	1,263	5,318,808,662
Stem (short d)	997.1	9.5	7,936,023	1,232	7,912,613,286
Root	1,060	8.4	5,272,384	1,389	5,591,241,404

### Genome size and characteristics

**Figure 1:**
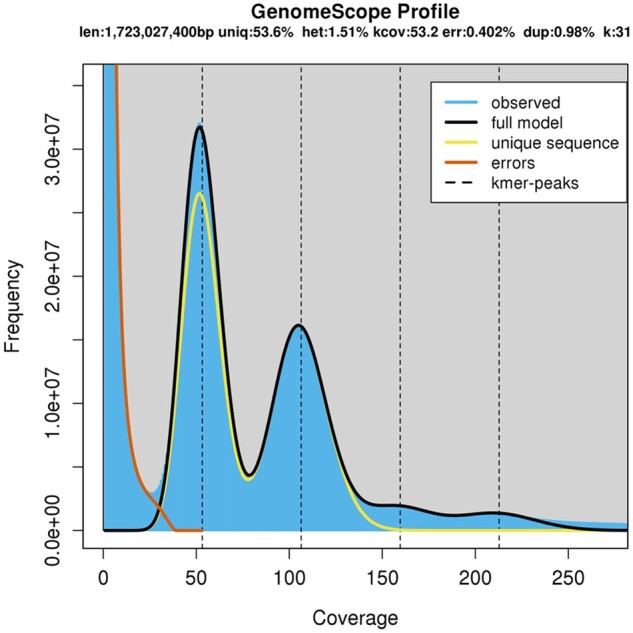
A k-mer (*K* = 31) distribution based on the illumina reads, modeled and visualized using genomescope.

k-mers (*K* = 31) were extracted from the paired-end HiSeq Illumina reads, counted using Jellyfish v2.2.10 (Marçais and Kingsford 2011) and analyzed with GenomeScope (Vurture *et al.* 2017) to estimate the genome haploid length, heterozygosity, and repeat content. The analysis converged and estimated a haploid genome size of 1.72 Gb, a heterozygosity of 1.51% (this value ranges from ∼0% to 2% (Vurture *et al.* 2017)) and marked 53.6% of the genome as unique (Figure  1). This indicates that the genome is repetitive and highly heterozygous. The haploid genome size of the chrysanthemum diploids has been estimated between 2.90 ± 0.03 Gb for *C. seticuspe* (Hirakawa *et al.* 2019) and 3.24 Gb for *C. nankingense* (Song *et al.* 2018) using flow cytometry. Previous genome size estimate of a *C. makinoi* (Nakano *et al.* 2019) suggested that the genome was approximately 10% larger than *C. seticuspe*, or approximately 3.19 Gb. The Genome Size Asteraceae Database estimates an average 1C of 3.82 Gb for chrysanthemum using flow cytometry, though this is likely an overestimation as the median is 3.1 Gb (Garnatje *et al.* 2011). It is known that sequence-based genome estimation methods underestimate genome size (Pflug *et al.* 2020) with GenomeScope being particularly sensitive to the k-mer count cut-off parameter (Vurture *et al.* 2017). This parameter is meant to distinguish repetitive sequences from organelle sequences, so that the repetitive k-mers are used to calculate the genome size while organelle k-mers are discarded, but this becomes impossible if the repetitive sequence k-mers are as abundant as the organelle k-mers (Vurture *et al.* 2017). With the high level of heterozygosity indicated by GenomeScope and confirmed with later analyses, it would be difficult to distinguish these k-mers from each other, resulting in many of the repetitive region k-mers also being discarded and producing a substantially underestimated genome size. We expect a true genome size closer to the previous cytometry predictions of 3.19 Gb (Nakano *et al.* 2019).

### Genome assembly and quality

After initial assembly with SMARTdenovo (Ruan *et al.* 2017) we had 39,105 contigs, spanning 4.1 Gb, with an N50 of 139.2 kb. Purge Haplotigs (Roach *et al.* 2018) produced a flattened assembly of 15,236 contigs, spanning 3.1 Gb, with an N50 of 255.8 kb. After two rounds of polishing with ntEdit v0.9 (Warren *et al.* 2019) using Illumina data, the assembly size was 3.1 Gb and made up of 15,226 contigs, with an N50 of 258.2 kb.

To scaffold the contigs, maps were generated using Hi-C and Chicago proximity ligation methods. This method generated 4254 scaffolds, covering a total length of 3.1 Gb, with an N50 of 168.9 Mb. The assembly was further superscaffolded into pseudochromosomes using ALLMAPS v0.9.14 (Tang *et al.* 2015) using a genetic map from a hexaploid *C. moriflorium* Ramat. (van Geest *et al.* 2017a). This resulted in a final assembly that was 3.1 Gb long and scaffolded into nine pseudochromosomes, with an N50 and L50 of 330.0 Mb and five scaffolds, respectively (Table  2).

**Table 2:** *C. makinoi de novo* genome assembly metrics estimated using QUAST

Assembly	*C. makinoi* V1.0 (9 chrs + chr0)
# Ns per 100 kbp	89.51
# contigs/scaffolds	10
Total length	3,113,668,257
N50	330,012,911
N75	317,988,395
L50	5
L75	7
Largest contig/scaffold	376,468,909
GC content (%)	36.01

The unplaced contigs were curated before being placed into chromosome 0 using the classification engine Centrifuge v1.0.4 (Kim *et al.* 2016). Of the 4206 unplaced contigs, 824 were marked as coming from a non-eukaryote source and removed. The Illumina reads were also aligned back to all the contigs using Minimap2 v2.17 (Li 2018) and, then, their coverage was assessed using Qualimap v2.2.1 (Okonechnikov *et al.* 2016). Contigs with a coverage higher than one standard deviation from the average were removed. This resulted in a final set of 3337 contigs, covering a total of 198.3 Mb, which were placed into chromosome 0.

BUSCO scores, which provide a set of universal single-copy orthologs, were also used to assess the completeness of the assemblies (see Table  3). Using the Embryophyta odb10 set with BUSCO v4.0.5 (Simão *et al.* 2015), the final assembly had a complete BUSCO score of 92.1% indicating a high overall quality. A full breakdown of the BUSCO score can be seen in Table  3.

**Table 3:** Output from the Busco analysis pipeline to assess gene complement completeness

BUSCO term	V1.0
Complete (%)	92.1
Complete and single copy (%)	83.8
Complete and duplicated (%)	8.3
Fragmented (%)	1.8
Missing (%)	6.1
Total	1375

For comparison, the exclusively short-read-based assembly of *C. seticuspe* had a total length of 2.722 Gb, with 354,212 contigs, an N50 of 44.7 kb, and the BUSCO score of 88.8% (Hirakawa *et al.* 2019). The *C. nankingense* assembly had a total length of 2.527 Gb, with 24,051 contigs, an N50 of 130.7 kb, and the BUSCO score of 92.7% (Song *et al.* 2018)*.* Thus, we were able to produce a substantially more contiguous assembly without sacrificing completeness.

### Repetitive regions

Using RepeatModeler (Flynn *et al.* 2020), 80.04% of the genome was marked as repetitive. Large genomes have accumulated repeats (Kelly and Leitch, 2011) and the k-mer analysis already indicated we were dealing with a largely repetitive genome. Of the 6799 identified repeat families in *C. makinoi*, 76.6% were identified as long terminal repeats (LTRs). Of the LTRs, 27.1% could be identified as *Copia* and 7.4% as *Gypsy*. A similar analysis in *C. nankingense* marked 69.6% of their assembly as repetitive and found LTRs to make up 67.7% of the identified tandem repeats, with 36.5% being *Copia* and 30.9% being *Gypsy* (Song *et al.* 2018). The lower rate of repetitiveness and identified LTRs in *C. nankingense* may be due to the difference in contiguity, with *C. nankingense* consisting of over 24,000 contigs (Song *et al.* 2018) to our 9 pseudochromosomes and 3337 unplaced contigs, as it has been shown that more complete genome assemblies will identify more LTRs (Ou *et al.* 2018). Analysis of various Asteraceae has shown fluctuations between members in relative abundance of *Copia vs* *Gypsy*, with sunflower (*Helianthus annuus*) amplifying *Gyspy* over *Copia* (Cavallini *et al.* 2010; Buti *et al.* 2011; Natali *et al.* 2013; Giordani *et al.* 2014; Badouin *et al.* 2017) while horseweed (*Conyza canadensis*) and globe artichoke (*Cynara Cardunculus* var. scolymus) showed the reverse (Peng *et al.* 2014; Scaglione *et al.* 2016). Earlier studies with *C. nankingense* and *C. seticuspe* (syn. *C. boreale*) suggested that in chrysanthemum the abundances of *Copia* and *Gypsy* were similar, with *Copia* being slightly more abundant and undergoing amplification slightly earlier (Song *et al.* 2018; Won *et al.* 2018a), but our results suggest that, at least in *C. makinoi*, there is a more substantial difference in abundance, like that seen in horseweed and globe artichoke. A systematic analysis of a variety of chrysanthemum species at various ploidy levels should be undertaken to gain better insight as these repeat types are a known driving force of plant genome evolution (Todorovska 2007).

### Transcript annotation

Each algorithm in the Funannotate (Palmer 2017) pipeline produced a set of *ab initio* gene models (see Supplementary Table S2). The evidence for each gene model was weighed using an EVM approach and identified 95,064 *ab initio* predicted gene models. This is higher than the plant average of 36,795 (Ramírez-Sánchez *et al.* 2016) but could be explained by the presence of pseudogenes (Xiao *et al*. 2016). Other Asteraceae including *Artemisia annua* (63,226 gene models; Shen *et al.* 2018), sunflower (52,232 gene models; Badouin *et al.* 2017), *Mikania micrantha* (46,351 gene models; Liu *et al.* 2020), *C. seticuspe* (71,057 gene models; Hirakawa *et al.* 2019), and *C. nankingense* (56,870 gene models; Song *et al.* 2018) also have substantially more than the average number of gene models. To investigate this further, an analysis of the structure and length of the annotated genes was also performed. The genes had an average coding sequence length of 876 bp and a maximum of 12,735 bp. This is shorter than the average plant gene length of 1308 bp but within the first quartile of average plant gene length (Ramírez-Sánchez *et al.* 2016). In line with the finding that plants tend to have less exons per protein than other organisms (Ramírez-Sánchez *et al.* 2016), 15.9% of the genes in *C. makinoi* were found to consist of a single exon. The average intron length within our gene set was found to be 446 bp, with a range of 11–19,668 bp and a median of 140 bp. This indicates that the majority of introns are relatively small. The distribution is similar to what has been found in maize (which had a mean of 516 bp and a median of 146 bp; Schnable *et al.* 2009).

Another explanation for the predicted genes being more abundant then is that average in plants could be due to ancient genome duplication. It has previously been reported that there was an ancient whole-genome triplication (WGT-γ) in dicotyledons (approximately 122–164 MYA) and another whole-genome triplication (WGT-1), before the split between asterids I and II, approximately 53–62 MYA (Badouin *et al.* 2017; Won *et al.* 2017; Liu *et al.* 2020). In addition, an analysis of the synonymous substitution rates of the paralogous and orthologous genes of the transcriptome assemblies of the hexaploid *C. morifolium* Ramat. and wild Korean diploid *C. boreale* revealed a whole-genome duplication or triplication event specific to chrysanthemum (Won *et al.* 2017). The assembly and annotation of more high-quality chrysanthemum genomes will help to clarify the genus’s evolution and its contributions to gene abundance.

**Figure 2:**
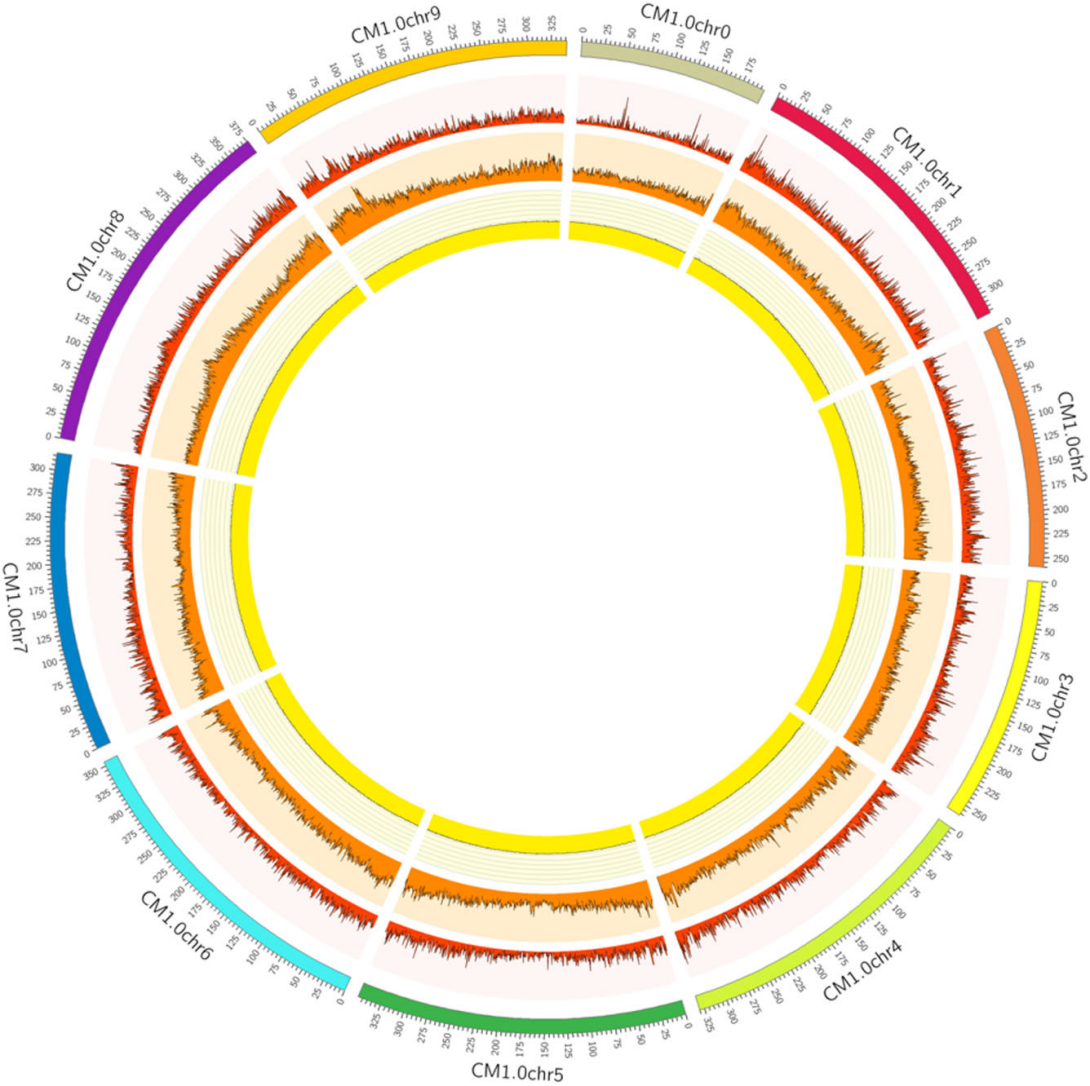
Circos plot showing the pseudomolecules (outer ring), gene density (red ring), repetitive element content (orange ring), and gc content (yellow ring) with a bin size of 500 kb.

Typically transposable elements accumulate in the centromeric and pericentromeric regions as they establish, maintain, and stabilize the centromeres of eukaryotes (Klein and O’Neill 2018). Thus, one can estimate the centromeric region of a chromosome based on a low gene density (Figure  2; red ring) and high repetitive sequence density (Figure  2; orange ring) but this pattern is not visible in *C. makinoi* as both the genes (red ring) and repetitive sequences (orange ring) are evenly distributed across. In fact, instead of clustering by region, the repetitive sequence density in *C. makinoi* has a positive Pearson correlation value of 0.60 with gene density. A possible explanation for this correlation is that chrysanthemum, like other Asteraceae, has LTRs driving a lot of diversity (Wang *et al.* 2014). Each LTR family has its own distribution characteristics in plant genomes (Chen 2007; Zhang *et al.* 2014) and LTRs make up 76.6% of the identified repeat families in *C. makinoi*. The sheer volume of the LTRs that distribute in gene-rich areas could be overwhelming the signal of repetitive sequences with a centromeric/pericentromeric preference. This is further supported by the previous work on repetitive elements in *C. seticuspe* (syn. *C. boreale*) that found, using optical techniques, a strong enrichment for LTRs and that the majority of repetitive sequences identified did not show a preference for centromeric or peri-centromeric regions (Won *et al.* 2018a).

Blast2Go (Götz *et al.* 2008) was used to functionally annotate the final gene model set. From our predicted gene models, 11.0% were assigned a putative functional label and 2.9% an enzyme code. Looking at the GO-level distribution, the majority of the gene models that were annotated as relating to a biological process (P) or molecular function (F) could not be identified to a high level of specificity, except the cellular component (C) annotated genes (see Supplementary Figure S3). This means that Blast2Go struggled to be more specific about the function of the identified biological process genes beyond, *i.e.*, “nitrogen compound metabolic process” but could get much more specific with the cellular component annotated genes.

## Conclusion

Having assembled the most complete and contiguous chrysanthemum genome available to date we have made an important step forward in our understanding of the genomics of this complex and important ornamental crop. This reference will provide a guide for further research in chrysanthemum breeding traits, origin, and strategies for assembling related higher ploidy varieties. This genome can act as a reference to assist in the ordering of other diploid chrysanthemum sequences as well as help to reduce the complexity of assembly in closely related polyploids as has been done in several other species (Lukaszewski *et al.* 2014; Li *et al.* 2015; Bertioli *et al.* 2016; Kyriakidou *et al.* 2018; Edger *et al.* 2019).

## Data availability

The final assembly and annotation files for *C. makinoi* Matsum. et Nakai (Japanese name: Ryuunougiku) No. JP131333 are available for download at www.chrysanthemumgenome.wur.nl/, along with a genome browser. All the raw data as well as the assembly and annotations files can also be found at ENA under PRJEB44800. The plant accession is available through the NARO Genebank.

##  

Supplementary material is available at *G3* online.

## Supplementary Material

jkab358_Supplementary_Figure1

jkab358_Supplementary_Figure2

jkab358_Supplementary_Figure3

jkab358_Supplementary_Tables-Captions
