# Supplementary figures and images for: De novo whole-genome assembly of Chrysanthemum makinoi, a key wild chrysanthemum
Source: G3 (Bethesda). 2021 Oct 13;12(1):jkab358. doi: 10.1093/g3journal/jkab358 (PMC8727959; doi:10.1093/g3journal/jkab358)

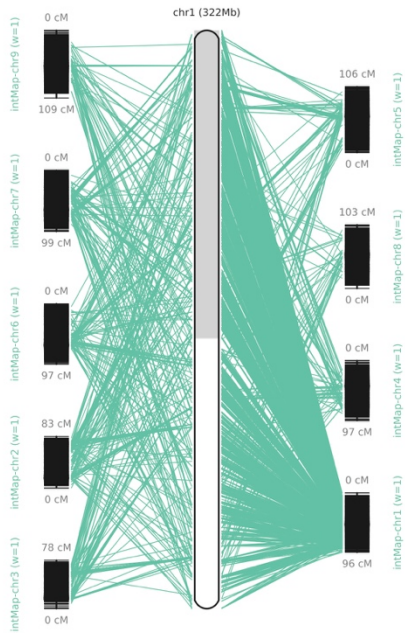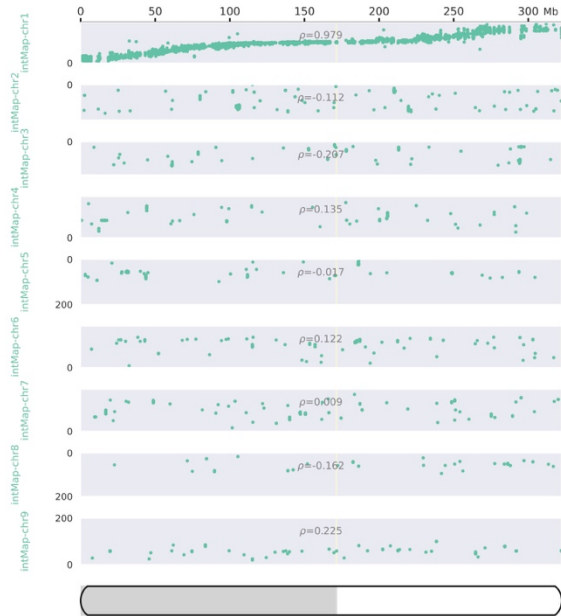

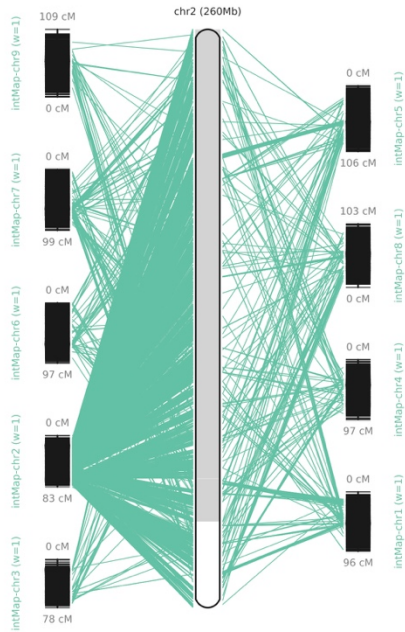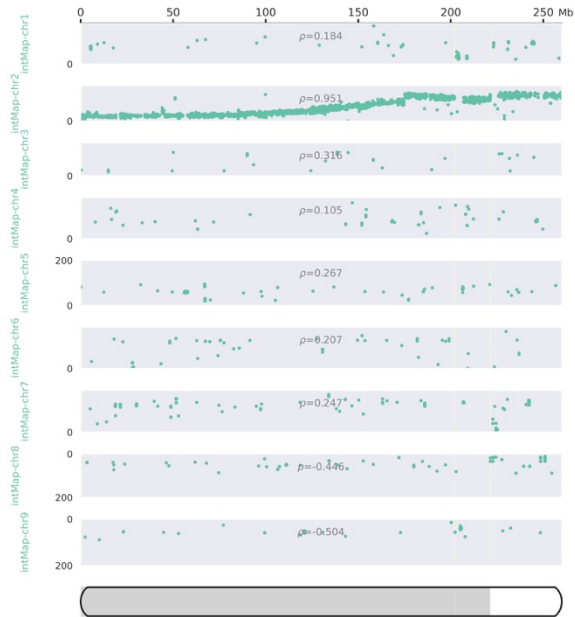

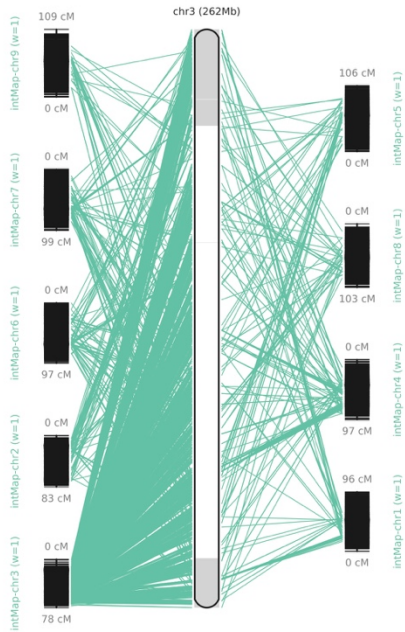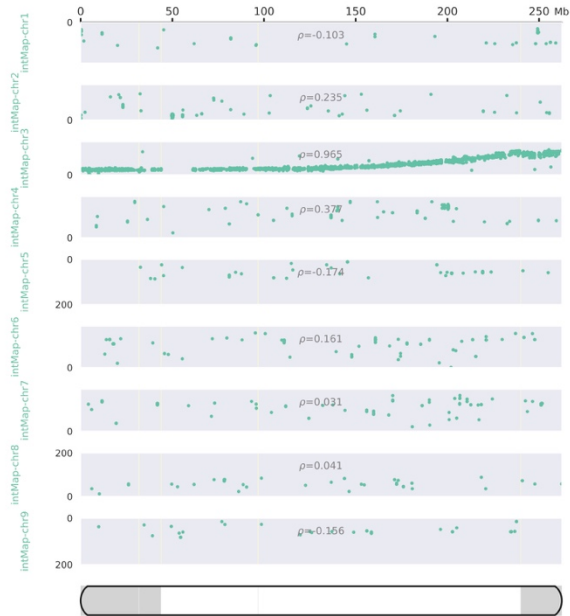

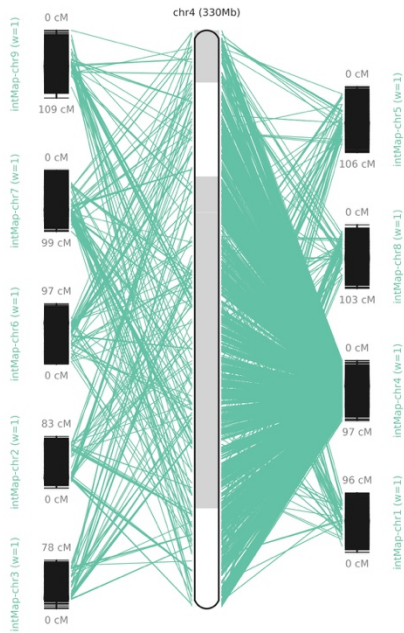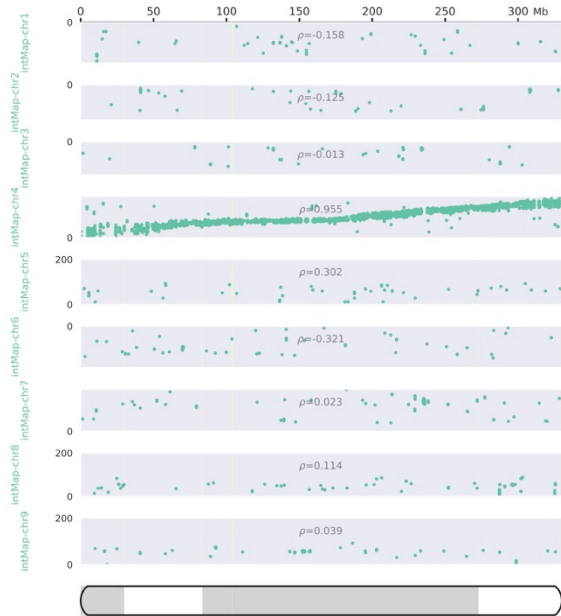

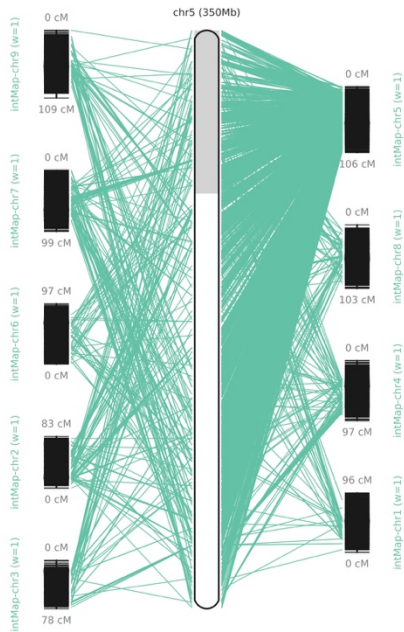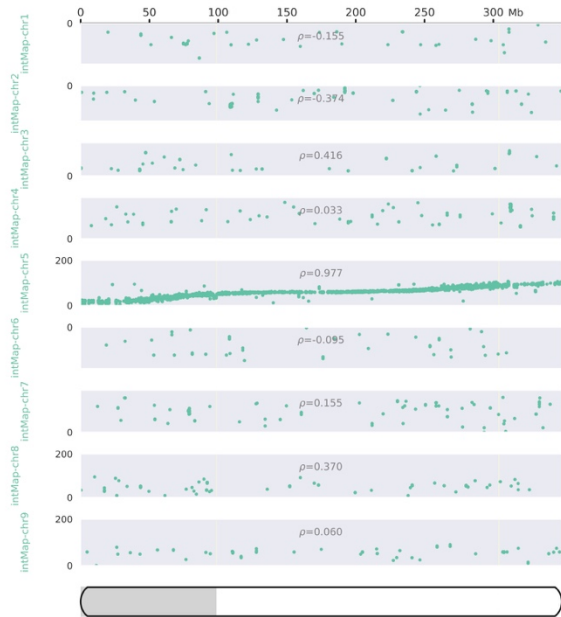

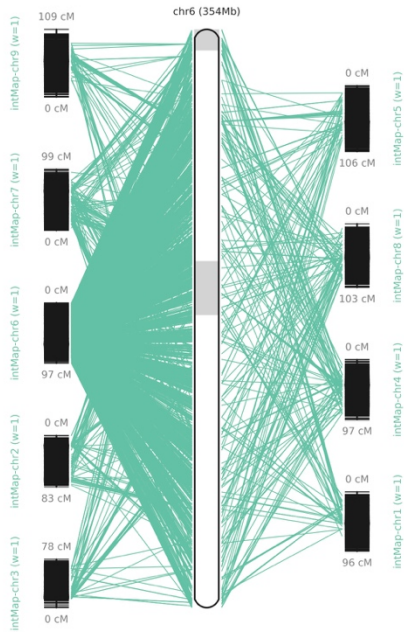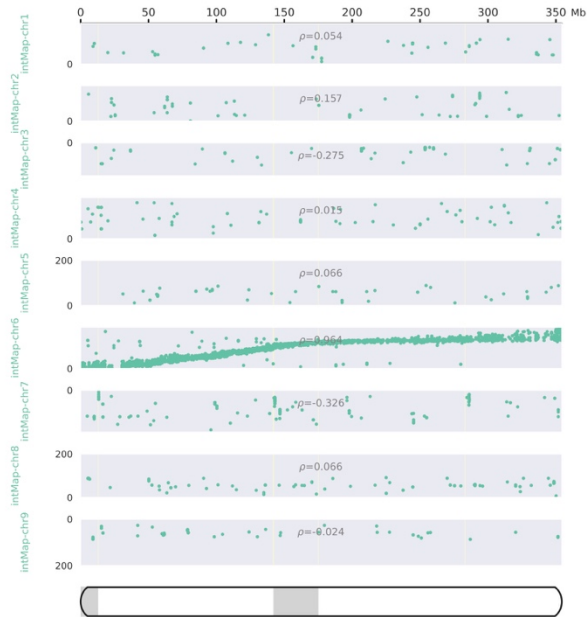

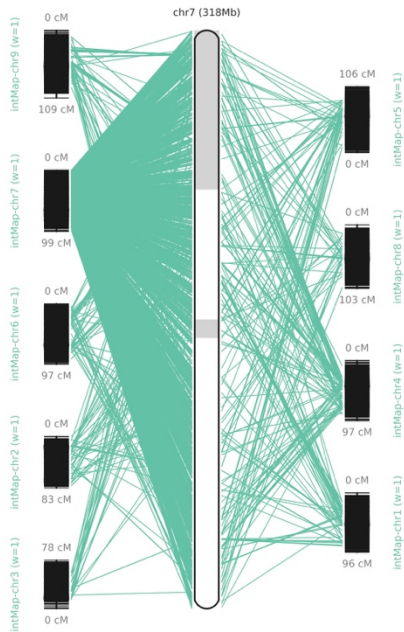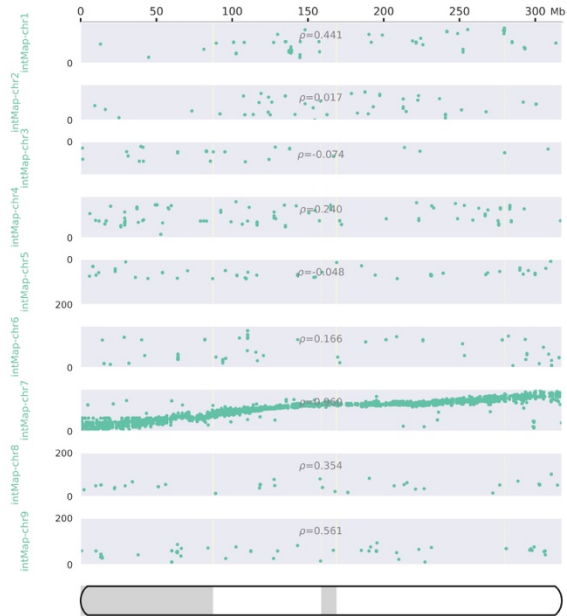

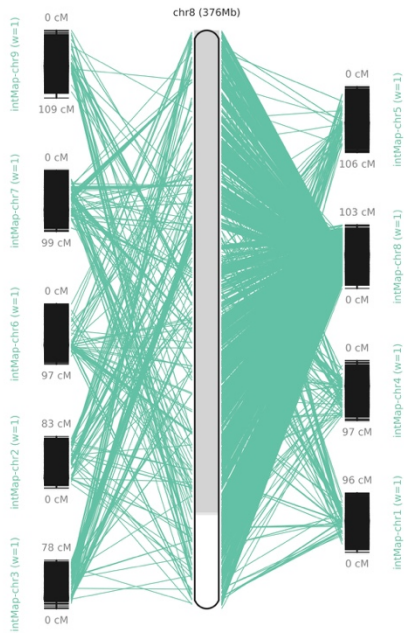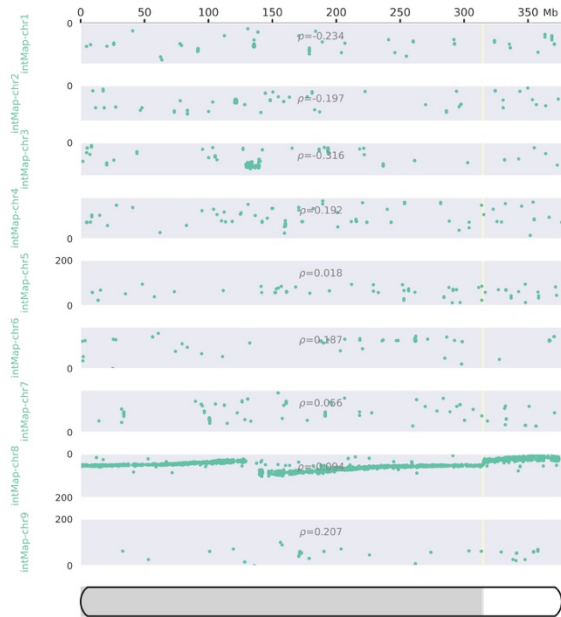

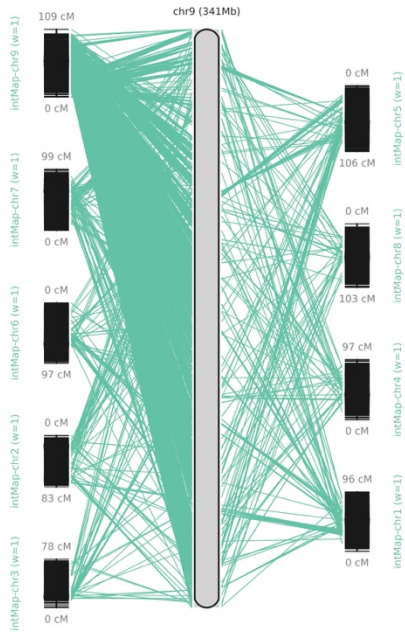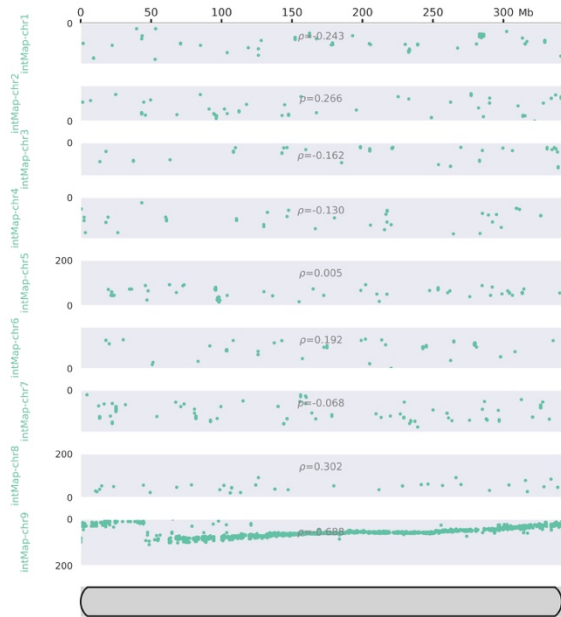

Supplement: jkab358_Supplementary_Figure1 [file jkab358_supplementary_figure1.pdf]

A

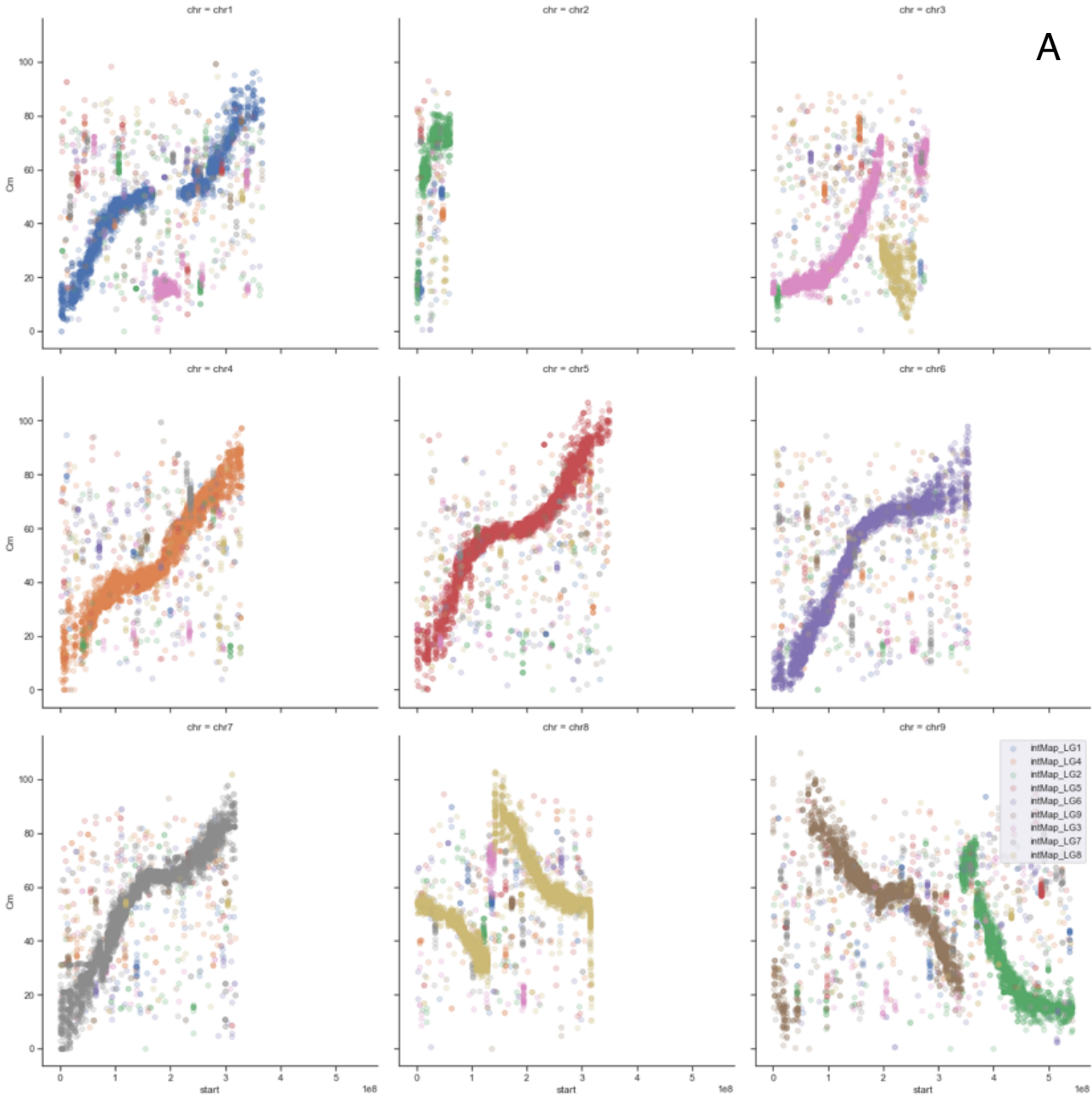

B

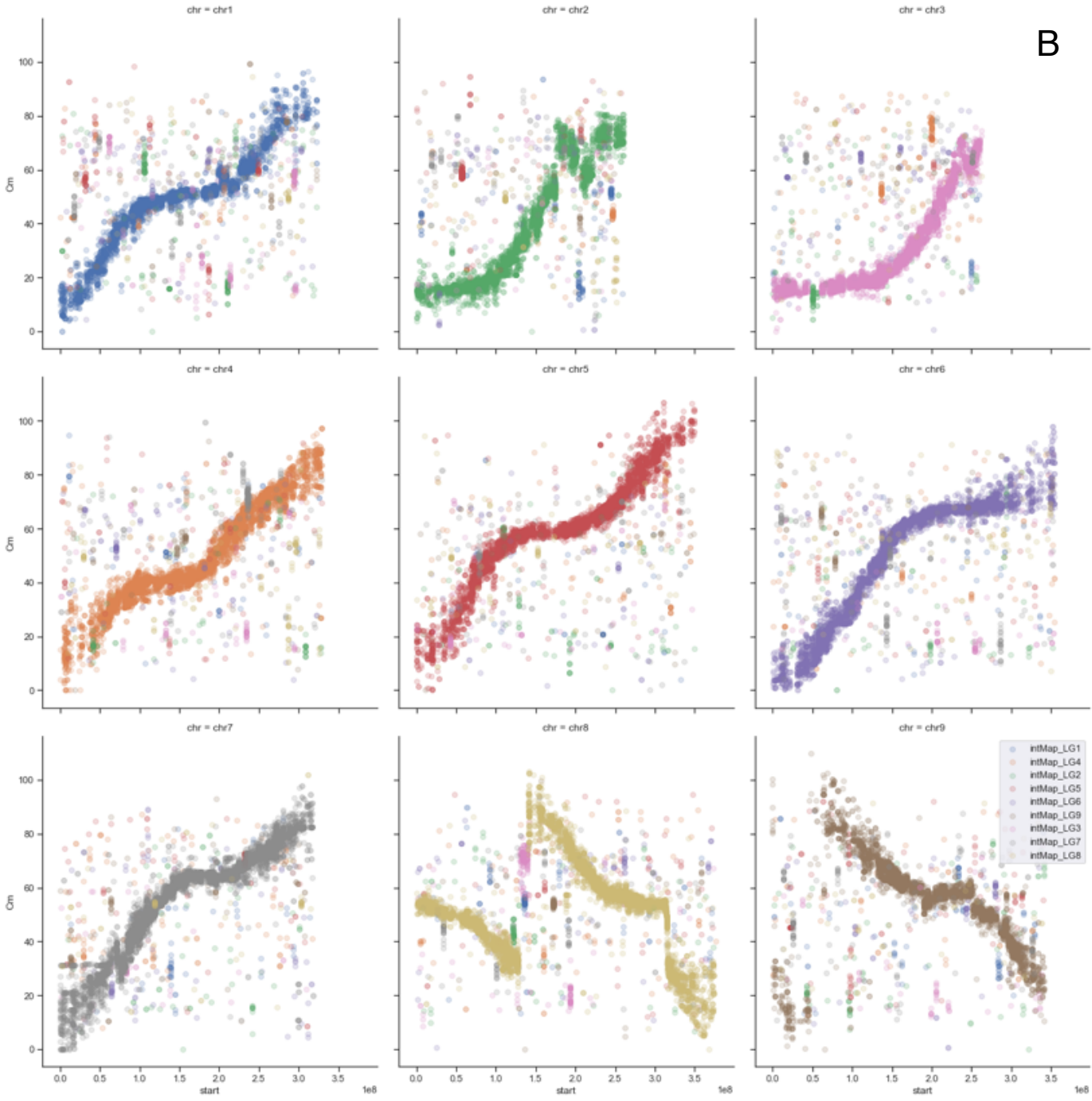

Supplement: jkab358_Supplementary_Figure2 [file jkab358_supplementary_figure2.pdf]

# GO-Level Distribution

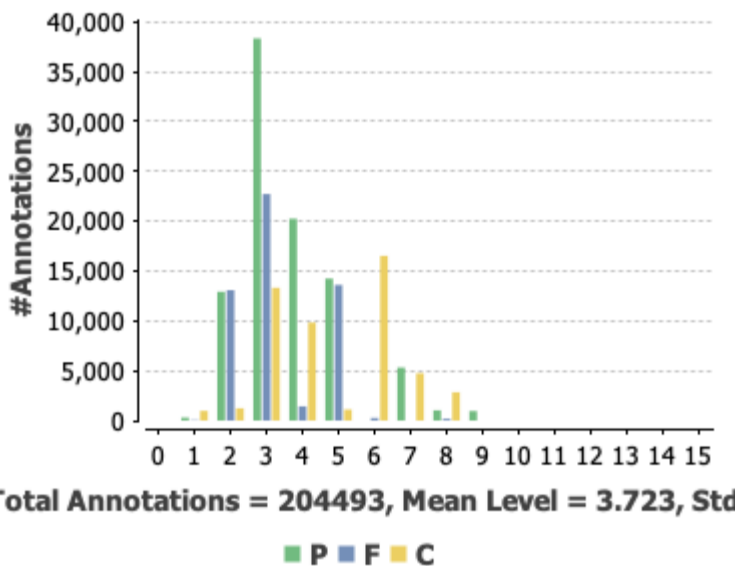

Supplement: jkab358_Supplementary_Figure3 [file jkab358_supplementary_figure3.pdf]
