# Supplementary material for: De novo whole-genome assembly of Chrysanthemum makinoi, a key wild chrysanthemum
Source: G3 (Bethesda). 2021 Oct 13;12(1):jkab358. doi: 10.1093/g3journal/jkab358 (PMC8727959; doi:10.1093/g3journal/jkab358)
Supplement: jkab358_Supplementary_Tables-Captions [file jkab358_supplementary_tables-captions.docx]

# SUPPLEMENTARY TABLES AND FIGURES

TABLE S1 ALLMAPS SUMMARY BETWEEN THE HEXAPLOID INTEGRATED GENETIC MAP AND C. MAKINOI SCAFFOLDS THAT DETAILS THE MARKER STATS FOR THE SEQUENCES THAT WERE ANCHORED, ONLY ORIENTED OR UNPLACED

|  | **Anchored** | **Oriented** | **Unplaced** |
| --- | --- | --- | --- |
| Markers (unique) | 48 482 | 48 393 | 910 |
| Markers per Mb | 16.6 | 16.6 | 4.0 |
| N50 Scaffolds | 8 | 8 | 0 |
| Scaffolds | 81 | 63 | 4 238 |
| Scaffolds with 1 marker | 8 | 0 | 105 |
| Scaffolds with 2 markers | 7 | 5 | 62 |
| Scaffolds with 3 markers | 2 | 2 | 28 |
| Scaffolds with ≥4 markers | 64 | 56 | 72 |
| Total bases | 2 914 525 386 (92.7%) | 2 912 184 103 (92.6%) | 229 073 915 (7.3%) |

FIGURE S1 ALLMAPS CHROMOSOME RECONSTRUCTION PLOTS OF C. MAKINOI USING A HEXAPLOID INTEGRATED GENETIC MAP, WHERE THE LEFT PLOT CONTAINS A SIDE BY SIDE ALIGNMENT OF CHROMOSOME AND LINKAGE GROUPS AND THE RIGHT SCATTERPLOT, THE PHYSICAL VS MAP LOCATIONS OF THE MARKERS. THE PHYSICAL LOCATIONS OF THE MARKERS WERE DETERMINED USING BLAST, USING THE BEST HIT. A SMALL SUBSET OF THE OF MARKERS WERE INCORRECTLY PLACED BUT THE HIGH DENSITY OF THE MAP PROVIDED AMPLE COVERAGE FOR ALLMAPS TO COME TO THE CORRECT CONSENSUS ANCHORING.

A

FIGURE S2 ALLMAP VISUALIZATION OF PSEUDOCHROMOSOME ASSEMBLIES AGAINST A HEXAPLOID GENETIC MAP BEFORE (A) AND AFTER (B) ONT VERIFIED BY-HAND CORRECTIONS. EACH PLOT REPRESENTS A C. MAKINOI PSEUDOCHROMOSOME WHILE THE COLOURS REPRESENT LINKAGE GROUPS FROM THE HEXAPLOID GENETIC MAP (AS INDICATED BY THE GREY BOX LEGEND IN THE BOTTOM RIGHT CORNER). PLOTS SHOW PREDOMINANT SYNTENY BETWEEN PSEUDOCHROMOSOME ASSEMBLIES AND LINKAGE GROUPS.

B

TABLE S2 NUMBER OF GENE MODELS PRODUCED BY EACH ALGORITHM AS PART OF THE FUNANNOTATE PIPELINE.

| **Algorithm** | **Number of Models** |
| --- | --- |
| Genemark | 212 885 |
| Augustus | 81 180 |
| High Quality Augustus | 23 894 |
| GlimmerHMM’ | 977 715 |
| Snap | 557 986 |

FIGURE S3 GO-LEVEL DISTRIBUTIONS OF SPECIFICITY FROM THE C. MAKINOI ANNOTATIONS SORTED BY FUNCTION WHERE THE X-AXIS INDICATES THE GO LEVEL AND THE Y AXIS THE NUMBER OF ANNOTATIONS AT THAT LEVEL. THE GREEN ‘P’ REPRESENTS GO TERMS WITH A BIOLOGICAL FUNCTION, THE BLUE ‘F’ GO TERMS WITH A MOLECULAR FUNCTION AND THE YELLOW ‘C’ GO TERMS WITH A CELLULAR COMPONENT.
